# Intraoral Laser Welding (ILW) in Implant Prosthetic Dentistry: Case Report

**DOI:** 10.1155/2012/839141

**Published:** 2012-08-09

**Authors:** Carlo Fornaini, Elisabetta Merigo, Igor Cernavin, Gonzalo Lòpez de Castro, Paolo Vescovi

**Affiliations:** ^1^Oral Medicine and Laser-Assisted Surgery Unit, Dental School, Faculty of Medicine, University of Parma, 29017 Fiorenzuola d'Arda, Italy; ^2^School of Dental Science, Faculty of Medicine and Dentistry, University of Melbourne, Melbourne, VIC, Australia; ^3^Facultad de Medicina y Odontología, Universidad de Santiago de Compostela, Madrid, Spain

## Abstract

The aim of this clinical study was to describe the possibility of using the Nd:YAG laser device utilized in the dental offices to weld metals intraorally. The authors, before applying this technique “in vivo” on human subjects, tested the “in vitro” metal welding efficacy of dental Nd:YAG device firstly by interferometry, SEM, and EDS and subsequently by thermal camera and thermocouples in order to record temperature changes during the welding process on bovine jaws. Four implants were inserted in the edentulous maxillary arch of a 67 years old male patient. Immediately after that, a bar previously made by the dental technician was intraorally welded to the abutments by Nd:YAG laser (Fidelis Plus III, Fotona, Slovenia) with these parameters: 9.90 mJ, 1 Hz, 15 msec, 0.6 mm spot. Then the prosthesis was connected to the bar with four OT Caps. This clinical study, even if preliminary, suggests that laser welding technique may be intraorally used without side effects.

## 1. Introduction

In 1967 Gordon described the possibility of welding metallic portions of dental prosthesis using laser technology and this technique has been used since the 1970s in dental laboratories, rapidly demonstrating its advantages over traditional welding methods [1].

In fact, the procedure can be carried out directly on the master cast thereby eliminating the risk of inaccuracies and distortions due to the duplication of the model [2]. Furthermore as the heat source is a concentrated high-power light beam, the potential for distortion in the prosthetic components is minimized [3]. The process allows the possibility of welding adjacent to acrylic resin or ceramic parts with neither physical (cracking) nor colour damages [4] thereby reducing the work load by eliminating the necessity for remaking broken prosthetic or orthodontic appliances.

Laboratory tests have shown that laser-welded joints have a high reproducible strength [5].

In the literature two methods are described for use in intraoral welding, both based on the creation of an electric arc between two electrodes under an argon gas flux: the “Syncrystallization” [6] and the “Mondani Electrowelder” [7]. Both have some limits: they are effective only on titanium and its alloys, they cannot be used on patients with pacemaker, they cannot work with filler metal, and some of the energy necessary for the welding process spreads to the adjacent area (teeth, acrylic, ceramic, etc.).

The laser technique described in this paper is effective on all metals, can be applied either with or without filler metal and shielding gas, and due to the extremely small spot size of the beam (0.6 mm) is able to restrict the high temperature required to a very limited area.

It can be used on all patients and does not require a new and specific appliance, utilizing the same device currently available for oral treatments in the dental office.

The aim of this study was to describe the possibility of intraoral welding by using a Nd:YAG laser with a fiberoptic delivery system.

The laser used in this study was the Fidelis Plus III (Fotona, Ljubljana, Slovenia) which is a combination of two wavelengths (Er:YAG = 2940 and Nd:YAG = 1064); in this work only the Nd:YAG was used.

This device has the ability to emit in both the microsecond and millisecond range, and when used in the millisecond range the temperatures necessary for metal welding occur.

In initial tests a fiberoptic of 900 *μ*m was used with a dermatological handpiece which allowed a variable spot size, the smallest spot size being 3 mm. By decreasing the working distance an effective spot size of 1 mm was achieved.

Subsequently the Fotona company constructed a prototype allowing a 0.6 mm spot size and producing, with the same parameters, a fluence ten times greater than the original. Fotona projected also a contra-angle handpiece able to weld in the posterior oral areas.

When using a laser with a fiber optic delivery system, part of the energy is lost from the fiber, and for this reason, the output power was periodically evaluated using a power meter (Ophir Nova II, thermal head F150A) to check the stability of the laser emission.

By means of some tests on CoCrMo plates analysed by interferometric microscope [8], proper parameters were determined in order to produce a good welding process without distortions and fissures: output power 9.90 W; energy 9.90 J; frequency 1 Hz; spot size 0.6 mm; pulse duration 15 msec; fluence 3300 J/cm^2^; focal distance 30 mm.

To measure temperature increases in the areas surrounding the welded zone, bovine jaws were used [9].

U-shaped plates were attached to the molars and the temperature was monitored by recording teeth and bone with a thermal camera when welding the two plates. The experiment was repeated measuring temperature changes using four thermocouples placed within the pulp chamber, the sulcus, the bone, and the root.

Whilst a higher temperature rise was recorded in the pulp chamber for all of the twelve samples tested, it was less than 5.5°C, which is considered to be the critical value for pulpal vitality.

To evaluate the quality of the welding process made in a dental technician's laboratory (Rofin, Germany) and made using the Fidelis laser, the samples were compared and analysed by SEM and EDS technique.

The only significant difference observed between the groups under optical microscopy and SEM observation was between those welded without filler metal, in which a larger number of fissures were seen in the plates welded using the Fidelis plus III. The groups in which the filler was used showed minimal differences.

The EDS analysis in the welding zone showed a homogeneous composition of the CoCrMo alloy with no great differences between the groups [10].

In order to compare the mechanical properties of the samples welded using the Fidelis laser with those welded using Rofin, twenty steel round orthodontic wires (Filo Tondo Duro Leone. 030 C8080-30, Leone, Florence, Italy and 14′′ Straight Wire, Ortho Organizers Inc, San Marcos, CA, USA) were divided into five groups, the first without welding, the second and the third welded using the Fidelis laser with and without filler, and the fourth and the fifth using Rofin with and without filler.

The samples were analysed using the dynamic mechanical analysis (DMA) method, which can be simply described as the application of an oscillating force (stress) to a sample and analysing the material's response to that force using the Dynamic Mechanical Analyser Q800 (TA Instruments, New Castle, DE, USA).

In all cases no wires were broken, even under the maximum strength (20 N), and the modulus of elasticity analysis showed very little difference between the samples.

Once the above data had been collected and verified, in vivo tests on human patients were carried out [11].

The tests were performed according to the patients once the written informed consent was obtained and after examination of the used protocol by the Institutional Review Board (IRB) of our hospital in conformity with the Declaration of Helsinki.

## 2. Case Presentation

MC, 67 years old male came to our clinic for an examination. At the clinical observation, the man appeared edentulous on the upper arch where he wore a total removable prosthesis.

His problem was that the device was not stable and he had a great discomfort in speaking and eating.

Due to his economical condition, it was decided to stabilize his appliance by the insertion of four implants in the maxillary bone.

The medical history did not reveal any particular aspects and the patient confirmed he did not take any kind of medication.

So, impression of the upper arch was taken in order to construct a template for correctly positioning the implants.

The anaesthesia of the maxillary area was obtained by the injection of two phiales of 36 mg of mepivacaine hydrochloride + 0.018 mg of adrenaline (1 : 100.000) (Scandonest 2%, Septodont, France).

The insertion of the four implants 4.5 × 11 mm (AoN, Vicenza, Italy) was made flapless, with the aid of the template, and the preparation of the sites was done by surgical micromotor (Implantmed, W&H, Austria) in continuous physiological solution (Eurospital, Italy) irrigation.

**Figure 1:**
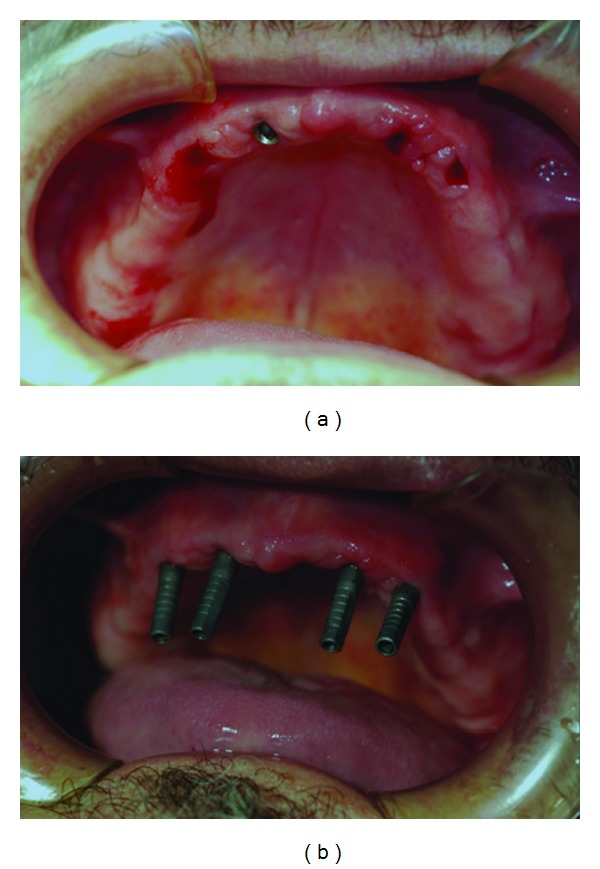
(a) Patient just after the four maxillary implants inserted. (b) Abutments screwed to the implants.

After the surgical procedure was performed (Figure 1(a)), four abutments were screwed to the implants (Figure 1(b)).

**Figure 2:**
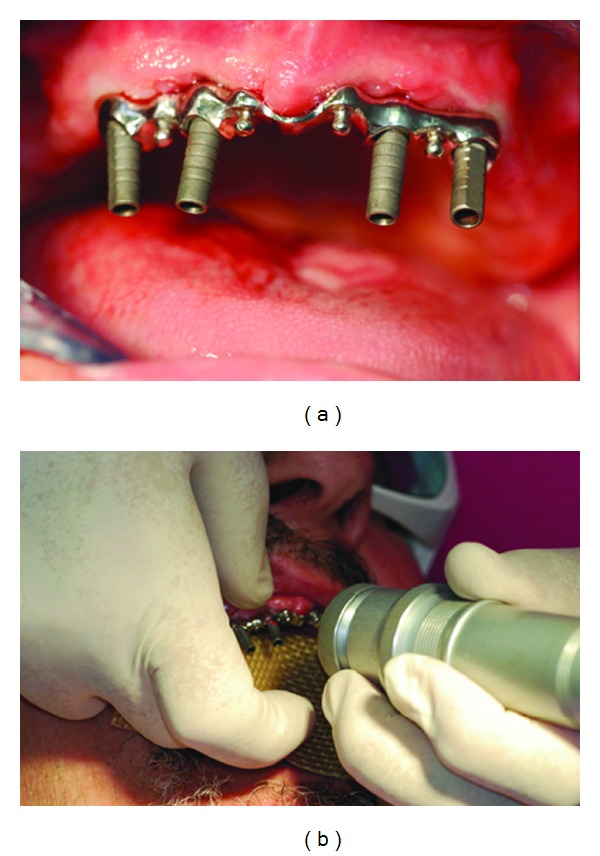
(a) Bar inserted in the abutments. (b) Intraoral laser welding.

Then, a bar previously constructed by the dental technician previewing the position of the implants by the maxillary arch impression was inserted in the four abutments (Figure 2(a)).

The bar was welded intraorally (Figure 2(b)) in order to fix the position, by dental Nd:YAG laser device (Fidelis Plus III) with these parameters: output power 9.90 W; energy 9.90 J; frequency 1 Hz; spot size 0.6 mm; pulse duration 15 msec; fluence 3300 J/cm^2^; focal distance 30 mm.

**Figure 3:**
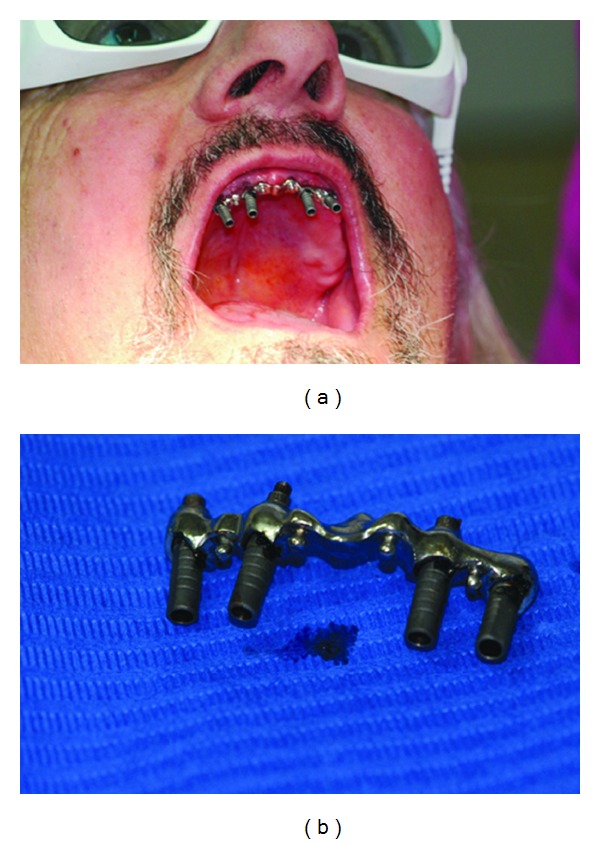
(a) The bar welded to abutments. (b) Bar and abutments removed from the mouth.

The whole intraoral welding procedure had a duration of 47 sec, and during it the patient said not to have felt any pain or discomfort (Figure 3(a)).

**Figure 4:**
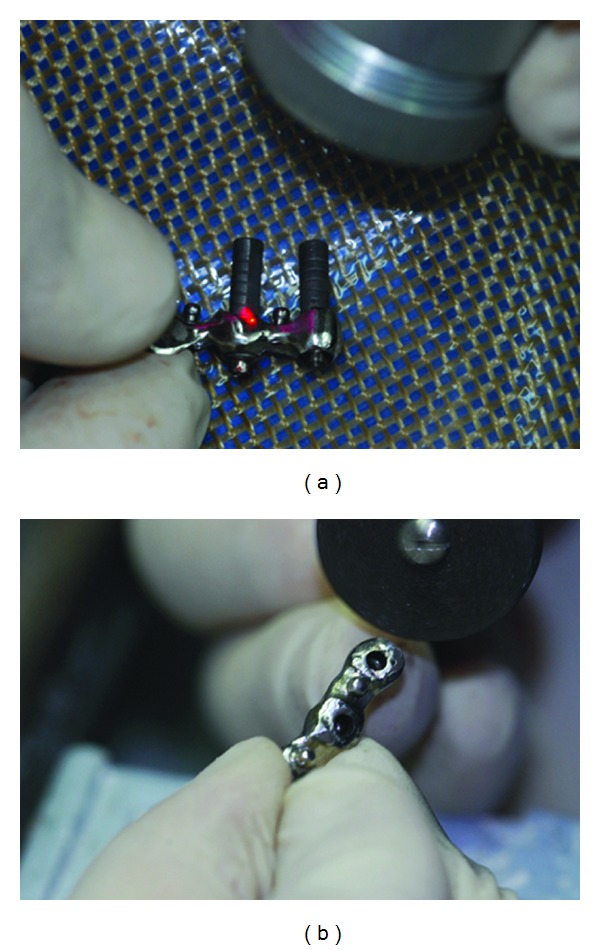
(a) The welding procedure extraorally completed by the same device. (b) The abutment cut by disc.

**Figure 5:**
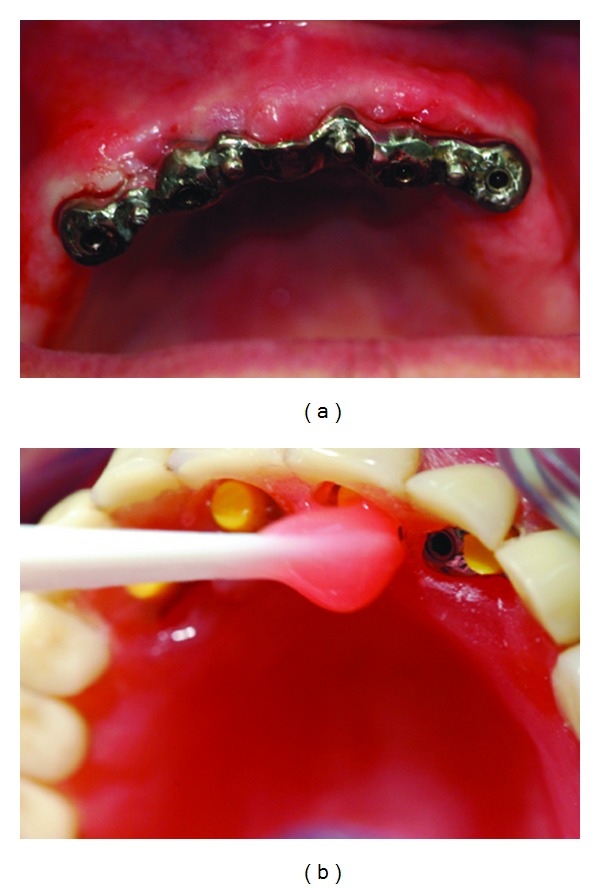
(a) The bar reapplied into the mouth. (b) The OT Caps fixed with acrylic to the prosthesis.

The bar was removed from the mouth with the abutments (Figure 3(b)) and the welding procedure was completed extraorally with the same device previously used (Figure 4(a)), the abutments were cut and polished, and then it was reapplied into the mouth (Figures 4(b) and 5(a)).

The prosthesis was then connected to the bar with four silicon OT Cap (Rhein 83, Italy) fixed by acrylic (Figure 5(b)).

The patient was checked after two days, seven days, and fifteen days, then monthly for six months, and during this period, no problems were noted.

## 3. Discussion

The results as presented above showed that it is possible to use a dental Nd:YAG laser (Fidelis Plus III, Fotona) to safely and effectively weld metals intraorally.

During the welding process the patient did not report any feeling of pain and discomfort.

The follow-up even after several months did not show any damages in the biological tissues. The welding process obtained by Fidelis was shown to be effective also after months, and even under the masticatory strength, no relapse was observed.

This preliminary study has shown the possibility of metal prosthodontic components intraoral welding without risk or discomfort to the patient thereby eliminating the costly and time-consuming process of impression taking with its inherent inaccuracies. The strength of the weld produced is not different to that produced by a dental technician laboratory laser.

Even if this clinical work must be considered as preliminary, it opens a new perspective on the utilization of laser technology in dentistry and therefore improving the quality of the results, reducing the operative time, and giving greater comfort to the patients.
